# Phthalate-Induced Fetal Leydig Cell Dysfunction Mediates Male Reproductive Tract Anomalies

**DOI:** 10.3389/fphar.2019.01309

**Published:** 2019-11-06

**Authors:** Yiyan Wang, Chaobo Ni, Xiaoheng Li, Zhenkun Lin, Qiqi Zhu, Linxi Li, Ren-Shan Ge

**Affiliations:** ^1^Department of Anesthesiology The Second Affiliated Hospital and Yuying Children’s Hospital of Wenzhou Medical University, Wenzhou, China; ^2^Center of Scientific Research, the Second Affiliated Hospital and Yuying Children’s Hospital of Wenzhou Medical University, Wenzhou, China

**Keywords:** phthalates, fetal Leydig cells, development, male, reproductive tract anomalies, testosterone

## Abstract

Male fetal Leydig cells in the testis secrete androgen and insulin-like 3, determining the sexual differentiation. The abnormal development of fetal Leydig cells could lead to the reduction of androgen and insulin-like 3, thus causing the male reproductive tract anomalies in male neonates, including cryptorchidism and hypospadias. Environmental pollutants, such as phthalic acid esters (phthalates), can perturb the development and differentiated function of Leydig cells, thereby contributing to the reproductive toxicity in the male. Here, we review the epidemiological studies in humans and experimental investigations in rodents of various phthalates. Most of phthalates disturb the expression of various genes encoded for steroidogenesis-related proteins and insulin-like 3 in fetal Leydig cells and the dose-additive effects are exerted after exposure in a mixture.

## Introduction

**Figure 1:**
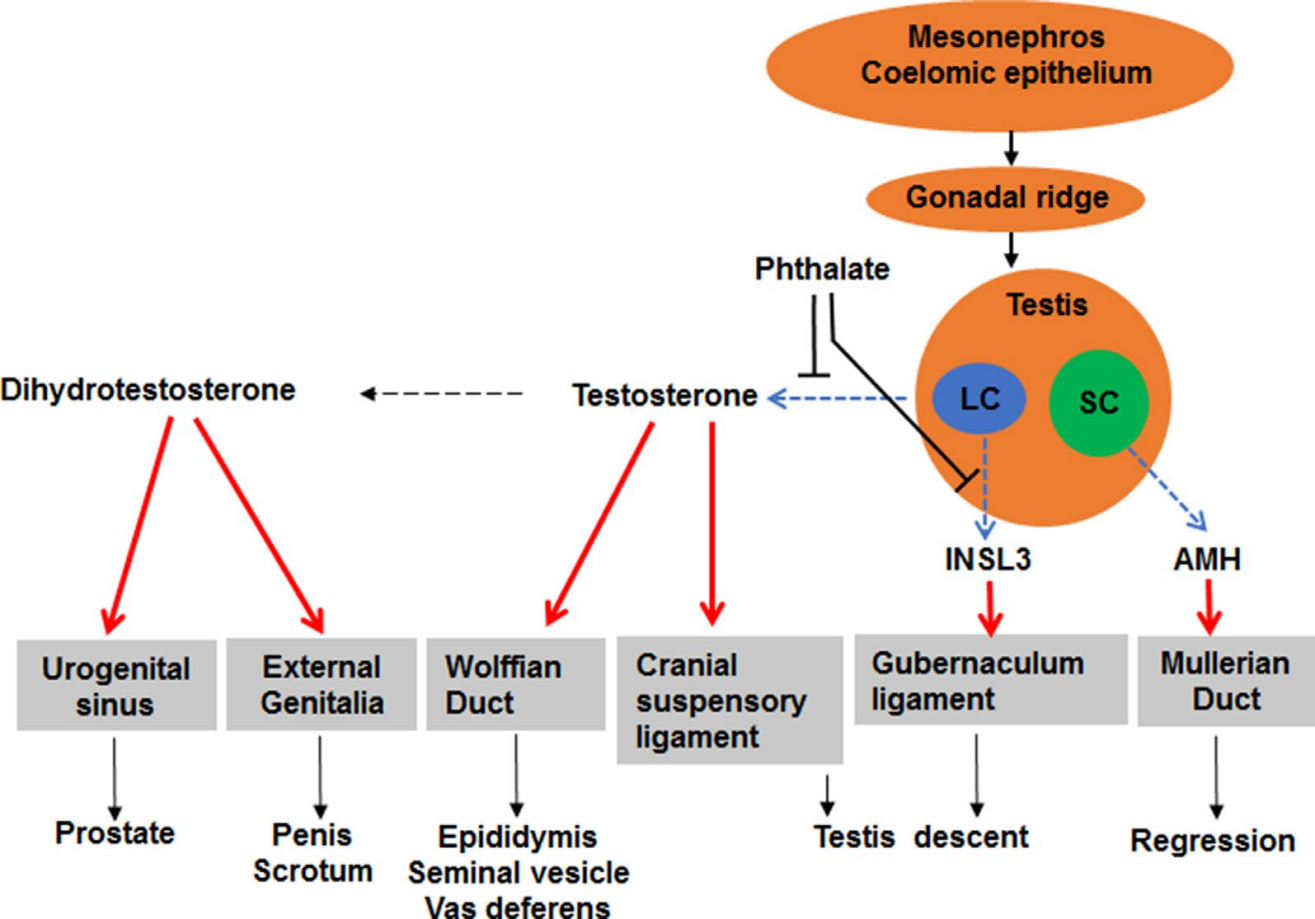
Illustration of sexual differentiation and phthalate action. The development of male reproductive tracts depends on androgen produced by fetal Leydig cells (LC) and anti-Müllerian hormone (AMH) produced by Sertoli cells (SC) after fetal gonad differentiation. Phthalate disrupts fetal Leydig cell development and function, thus lowering androgen and insulin-like 3, thereby causing male reproductive tract anomalies after birth.

Reports of decline in sperm counts of human adult males over the past 60 years and the reproductive tract anomalies in new-born baby boys exposed to the chemicals in the environment have generated much public concern (Moline et al., 2000). By acting as ligand-inducible transcription factors that activate or repress transcription of target genes, sex steroid hormone receptors control fundamental events in the embryonic development and sexual differentiation. Agonists and antagonists of the sex steroid hormone family of the nuclear transcription factors, androgen and estrogen receptors, are associated with the reproductive tract anomalies. Androgens are the predominant male reproductive hormones, and the primary steroid hormone, testosterone, is produced by the testicular Leydig cell, a cell type in the interstitial compartment of the testis (**Figure 1**). Testosterone can be converted into more potent androgen, dihydrotestosterone, either within Leydig cells or in peripheral tissues (**Figure 1**). Prenatal testosterone or dihydrotestosterone exposure stimulates sexual differentiation *via* promoting the generation of male reproductive tracts and postnatally they promote the development of the male sexual characteristics and maintain the male phenotype in adulthood (**Figure 1**). Testosterone is also converted to estradiol by the aromatase in the brain (Balthazart and Foidart, 1993; Callard et al., 1993; Roselli and Resko, 1993), resulting in activation of estrogen receptor-mediated pathways associated with male sexual behavior (Meisel and Sachs, 1994).

There is growing evidence that developmental anomalies in the male reproductive tract result from insufficient androgen action during embryonic development (Adami et al., 1994). Various environmental chemicals could cause this condition (Akingbemi and Hardy, 2001). One family vof these chemicals are phthalate esters (phthalates), which are widely used in the plastic industry and they are credited with the inhibition of androgen synthesis activity because they suppress androgen-stimulated sexual differentiation in rodents (Akingbemi and Hardy, 2001; Sharpe, 2001). For example, after exposure of prepubertal rats to di(2-ethylhexyl) phthalate (DEHP) *via* oral administration, no observed adverse effect level (NOAEL) and lowest observed adverse effect level (LOAEL) of lowering testosterone levels to be 1 mg/kg/day and 10 mg/kg/day, respectively (Akingbemi et al., 2001). Furthermore, there is a growing body of evidence showing that exposure to DEHP adversely affects Leydig cell steroidogenesis and sexual development in rodents (Gray et al., 2000; Parks et al., 2000; Akingbemi et al., 2001; Moore et al., 2001). Here, we review the effects of phthalates on fetal Leydig cell development and function and the possible adverse outcomes. Since many data were acquired from rats and humans, we focused our discussion in these two species.

## Leydig Cell Development and Function

### Generations of Leydig Cells

Leydig cells are the primary and only significant source of androgen in males (Dong and Hardy, 2004; Ye et al., 2017). Developmentally, Leydig cells appear as two distinct populations: fetal Leydig cells prenatally and adult Leydig cells during pubertal maturation in rodents (Huhtaniemi and Pelliniemi, 1992; Ge and Hardy, 2007; Ye et al., 2017). The first population exists in the fetal rodent testis. The primary function of androgen secreted by fetal Leydig cells is the stimulation of sexual differentiation and formation of male reproductive tracts. Postnatally, a second population develops during rodent puberty. Testosterone secreted by the second population in rodents stimulates secondary sex characteristics and maintains sperm production in adulthood (Huhtaniemi and Pelliniemi, 1992; Ge and Hardy, 2007; Ye et al., 2017). Then, androgen declines with age due to Leydig cell aging (Zirkin and Tenover, 2012). In humans, there is another population of Leydig cells referred as neonatal Leydig cells, which occur between fetal and adult Leydig cell populations. This population develops during the neonatal stage (Prince, 1990), due to the transient activation of the hypothalamic–pituitary–testicular axis at age of 6 months (Prince, 1990). This population of Leydig cells involutes or dedifferentiates rapidly. They secrete testosterone to possibly support hormonal imprinting of androgen-sensitive tissues (e.g., the liver, prostate, and hypothalamus) (Prince, 2001; Ye et al., 2017).

### Androgen and INSL3 Production and Regulation

The main function of Leydig cells is to synthesize androgen (Dong and Hardy, 2004; Ye et al., 2017). Androgen synthesis in the testis begins with a steroid substrate, cholesterol (**Figure 1**) (Rommerts and Brinkman, 1981). Cholesterol can not only be synthesized *de novo* (Hou et al., 1990) but also be transported into both fetal Leydig cells and adult Leydig cells from high density lipoprotein after its binding to scavenger receptor class B member 1 (SCARB1, encoded by *Scarb1* gene) (Saez et al., 1983; Zhang et al., 2015). Intracellular cholesterol in Leydig cells is required to be transported into the inner mitochondrial membrane by the steroidogenic acute regulatory protein (STAR, encoded by *Star* gene) (Stocco, 2000; Stocco, 2001) for the first-step catalysis by a cytochrome P450 enzyme, cholesterol side-chain cleavage enzyme (CYP11A1, encoded by *Cyp11a1* gene). CYP11A1 is a multiple enzyme complex, which converts cholesterol *via* a series of enzymatic reactions into steroid pregnenolone (Mast et al., 2010). Pregnenolone is thought to diffuse out into the neighboring smooth endoplasmic reticulum, where several critical androgen synthetic enzymes are present: 3β-hydroxysteroid dehydrogenase 1 (HSD3B1, encoded by *Hsd3b1* gene), cytochrome P450 17α-hydroxylase/C17, C20-lyase (CYP17A1, encoded by *Cyp17a1* gene), and 17β-hydroxysteroid dehydrogenase 3 (HSD17B3, encoded by *Hsd17b3* gene). HSD3B1 converts pregnenolone into progesterone and CYP17A1 converts progesterone into androstenedione (Wen et al., 2016) and HDS17B3 converts androstenedione into testosterone (Wen et al., 2016). Interestingly, HSD17B3 is mainly located in fetal Sertoli cells in mice (Shima et al., 2013). Thus, fetal Leydig cells primarily produce androstenedione, which is converted to testosterone by HSD17B3 in Sertoli cells and fetal Leydig cells together with Sertoli cells jointly contribute to testosterone production in fetal mouse testis (Shima et al., 2013).

The development of fetal Leydig cells is possibly regulated by thyroid hormone and estrogen. A perinatal hypothyroidism caused by a reversible 6-propyl-2-thiouracil from gestational day (GD) 15 to postnatal day 20 lead to a small testis in the neonatal period (Kobayashi et al., 2014). Estrogen has also been postulated to cause increase in human male reproductive developmental disorders (cryptorchidism and hypospadias) (Sharpe, 2003). Estrogen-mediated action on these disorders could be due to suppression of testosterone and INSL3 production by fetal Leydig cells and suppression of androgen receptor expression (Sharpe, 2003). Diethylstilbestrol, a potent estrogen, inhibits *Insl3* expression *via* down-regulating the expression of steroidogenic factor 1 (NR5A1), a critical transcription factor for fetal Leydig cells (Zhang et al., 2009a).

Phthalates have little or very weak estrogen-like activity *in vitro* (Harris et al., 1997). There is increasing evidence to show that phthalates disrupt androgen synthesis. However, di-phthalates and their mono-metabolites do not bind to the androgen receptor *in vitro* (Parks et al., 2000). Phthalates also affect other cell types in fetal testis (Lin et al., 2008a; Hu et al., 2009). Sertoli cells and peritubular cells are phthalate targets. After exposure to phthalates, there is focal testis dysgenesis due to the disrupted peritubular myoid cells and disturbed Sertoli cell organization with some Sertoli cells in the interstitium (Mahood et al., 2005; Mahood et al., 2006; Lin et al., 2008a; Hu et al., 2009). Another action of phthalate is the formation of multinuclear gonocytes in the fetal testis (Mahood et al., 2005; Li et al., 2014; Li et al., 2016).

### Fetal Leydig Cell Functions

The genetic X and Y chromosomes determine sex of an embryo at fertilization (Albrecht et al., 2003). However, the sexual differentiation of the fetus starts when the gonads differentiate (Schmahl et al., 2004). Initially, gonads are identical in XY and XX embryos, referred as the indifferent gonads (Voutilainen, 1992). Around GD12 in mice or GD14 in rats and week 6 in humans (Bizarro et al., 2003; Schmahl et al., 2004), a bipotential gonad is formed and it gradually develops into either a testis or an ovary (Sinisi et al., 2003). Although the exact origin of fetal Leydig cells remain unclear, they are believed to migrate from the mesonephros to the bipotential gonad (**Figure 1**) (Barsoum and Yao, 2009). About 24 h after existence of another somatic cell, the Sertoli cell, fetal Leydig cells start to differentiate in the interstitium in the mice and rats and this differentiating process is thought to be controlled by Sertoli cell-secreted factors, such as desert hedgehog *via* binding to its receptor (Yao et al., 2002) and platelet derived growth factor *via* binding to its receptor (Brennan et al., 2003) and aristaless-related homeobox (Miyabayashi et al., 2013). Fetal Leydig cells are formed in the testicular interstitium from GD12 in mice or GD14 in rats and they persist until after birth (Barsoum and Yao, 2009). Fetal Leydig cells may involute gradually with only a few cells persisting in the adult testis (Kerr and Knell, 1988). There is still controversy about fetal Leydig cell fates (Wen et al., 2016). A recent study demonstrated that fetal Leydig cells persist in adult mouse testis (Shima et al., 2015). However, their contribution to androgen production in the adult testis is minimal (Kerr and Knell, 1988). The primary function of fetal Leydig cells is the synthesis of androgen, which promotes the development of both the internal and external genitalia of the male fetus.

### Fetal Leydig Cell-Secreted Androgen Promotes the Development of External Genitalia

Between weeks 9–12 during human pregnancy, the genitalia becomes sex specific. In males, the genital tubercle grows longer and the urethral groove exists in the ventral aspects. The groove and urethral folds extend along the shaft of the elongating phallus and terminate in a solid plate of epithelial cells that extends into the glans penis. When the urethral folds fail to fuse, an abnormal location of the urethral opening occurs and a disease called hypospadias is formed. Although the exact mechanism of the formation of hypospadias remains unclear, development of the male penis depends on the action of androgen. In the genital tubercle, a testosterone-metabolizing enzyme, 5α-reductase type 2 (SRD5A2) is present (**Figure 1**) and it converts testosterone into the more potent androgen dihydrotestosterone. Dihydrotestosterone binds to androgen receptors in all cells of the genital tubercle to promote its growth.

### Fetal Leydig Cell-Secreted Androgen Promotes the Development of Internal Genitalia

During the period of the bipotential gonad, both sexes have identical urogenital tracts, consisting of two duct systems, the Wolffian duct and the Müllerian duct (**Figure 1**). In embryonic males, the Wolffian duct develops into the epididymis, vas deferens, and seminal vesicles, and the Müllerian duct regresses after the action of Sertoli cell-secreted anti-Müllerian hormone (**Figure 1**) (Vigier et al., 1989). In the embryonic females, the Müllerian duct develops into the fallopian tubes, uterus, cervix and upper third of the vagina and the Wolffian duct regresses (Sugawara et al., 1989) and the absence of androgen causes the Wolffian duct to regress and the absence of anti-Müllerian hormone lets the Müllerian duct unaffected (Sugawara et al., 1989).

### Fetal Leydig Cell-Secreted Insulin-Like 3 (INSL3) and Androgen Promote the Descent of Testis

In rodents, fetal Leydig cells secrete androgen and INSL3 (a peptide hormone for gubernaculum shortening) (Adham et al., 2000; Emmen et al., 2000; Koskimies et al., 2000), which cause migration of the testes within the abdomen (**Figure 1**). There are two phases of testis descent. Within the first phase, testes travel across the abdomen to the entrance of the inguinal canal. This phase requires both androgen and INSL3 (Huang et al.; Adham et al., 2000; Hughes and Acerini, 2008). Testosterone induces the dissolution of the cranial suspensory ligaments, which retains the testes in a perirenal position (**Figure 1**) (Van Der Schoot and Elger, 1992). Within the second phase, testes travel the inguinal canal into the scrotum and this phase is thought be androgen-related. Androgen stimulates the secretion of calcitonin gene related peptide, which causes contractions of the gubernaculum that guides the testes into the scrotum (Sugawara et al., 1986). In humans, recent several studies indicate that the developing epididymis to enlarge and hold the testis towards the developing scrotum during the process of descent (Hadziselimovic, 2017). Barteczko and Jacob (2000) described five phases of human testis descent: In phase I, early development of the gubernaculum occurs; in phase II, three parts of the gubernaculum-abdominal, interstitial, and subcutaneous are distinguished; in phase III, gubernaculum swells and the testis glides across the genital ducts.

### Regulation of Fetal Leydig Cell Development

Fetal Leydig cell development is regulated by many hormones and growth factors (Wen et al., 2016). Whereas pituitary-secreted luteinizing hormone (LH) is the primary regulator of Leydig cell differentiated function, initiation of steroidogenesis in fetal Leydig cells is independent of LH in rodents although LH-stimulated testosterone production increases gradually from gestation day 16 in mice (Baker and O'shaughnessy, 2001). Evidences to supporting this notion are: mice with a congenital deficiency in gonadotropin-releasing hormone (GnRH) resulting in a lack of exposure of the gonads to endogenous LH have functional fetal Leydig cells (O'shaughnessy and Sheffield, 1990). Without exogenous LH, these mice remain in a prepubertal, less undifferentiated state as evidenced by exhibiting very low levels of INSL3 (Balvers et al., 1998), which is a marker for fully differentiated fetal Leydig cells (Teerds, 1996; Balvers et al., 1998). Finally, Leydig cells differentiate in LH receptor knockout mice, although their numbers are considerably lower compared to control mice (Lei et al., 2001; Zhang et al., 2001). This observation suggests that factors other than LH are important in the regulation of the production of testosterone in the fetal testis. These factors could be insulin-like growth factor 1 (Mullaney and Skinner, 1991), desert hedgehog (Yao et al., 2002; Callier et al., 2014), activin (Archambeault et al., 2011), kit ligand (Tsikolia et al., 2009), SMOC1 and SMOC2 (Pazin and Albrecht, 2009), notch ligands (Tang et al., 2008), estrogen (Delbes et al., 2005), and platelet-derived growth factor AA (Brennan et al., 2003). LH is generally regarded (Huhtaniemi et al., 1981; Huhtaniemi et al., 1984; Benton et al., 1995) as a key regulator of fetal Leydig cell steroidogenesis at the late stage. In humans, the development of human fetal Leydig cells is dependent upon human chorionic gonadotropin (HCG) as shown by the fact that patients with an inactivating mutation of the LH receptor have severely reduced number of fetal Leydig cells and they are pseudohermaphrodites.

## Phthalate Exposure

### Phthalate Type and Exposure Levels

Phthalates are esters of phthalic acid linking with different alcohols with one (dimethyl phthalate, DMP) or more than one carbon numbers (such as ditridecyl phthalate, DITP, with 13 carbon atoms) in the alcohol moiety (**Table 1**). Phthalates are used as plasticizers in a wide range of polyvinyl chloride-based consumer products, e.g., food packaging products, medical devices, infant toys, construction materials, wires and cables, and conveyor belts. Phthalates of lower molecular weights (3-6 carbon alcohols) are being gradually replaced in numerous products in the Western countries by those of high molecular weights (greater than 6 carbons in their backbone) because of health concerns (Su et al., 2012). Among the production volume in the United States, diisononyl phthalate (DINP) had the highest production volume, followed by diisodecyl phthalate (DIDP) and DEHP, and then butyl benzyl phthalate (BBP), di-n-butyl phthalate (DBP), di-n-octyl phthalate (DOP) and diisobutyl phthalate (DIBP) (Su et al., 2012). DEHP is the preferred plasticizer in medical applications, which measures 8 ng/cm^3^ to 3 mg/cm^3^ in the indoor air (Weschler, 1984), and typical human exposure level is estimated at 30 µg/kg/day although occupational and clinical exposures may increase this level to 5 mg (Doull et al., 1999). The US health care industry alone uses more than 500 million intravenous bags each year. There is a public health concern that patients undergoing hemodialysis and blood transfusions are exposed to unusually high DEHP levels after it leaches out of polymer medical devices (e.g., intravenous bags, tubing, and blood bags). Thus, human exposure to DEHP is significant. In China, the economy remains largely based on traditional agriculture, and a close relationship was observed between the concentration of phthalates in soils and the consumption of plastic film used to wrap plants protectively and insulate the soil (Thulin et al., 1984). A survey results demonstrated that phthalates were ubiquitous pollutants in arable soils in China (Tian et al., 2018). The total concentrations of phthalates ranged from 0.89 to 10.03 mg kg^−1^ with a median concentration of 3.43 mg kg^−1^(Tilton et al., 2003). Among the phthalates, DEHP was the predominant form (Tilton et al., 2003). The correlation indicates that the application of agricultural plastic film might be a significant pollution source of phthalates in arable soils of China.

**Table 1:** Phthalate products and their structures.

Carbon number of alcohols	Common name	Acronym	Structural formula
1	Dimethyl phthalate	DMP	C6H4(COOCH3)2
2	Diethyl phthalate	DEP	C6H4(COOC2H5)2
3	Di-n-propyl phthalate	DPrP	C6H4[COO(CH2)2CH3]2
4	Di-n-butyl phthalate	DBP	C6H4[COO(CH2)3CH3]2
4	Diisobutyl phthalate	DiBP	C6H4[COOCH2CH(CH3)2]2
4/6	Butyl benzyl phthalate	BBP	CH3(CH2)3OOCC6H4COOCH2C6H5
5	Di-n-pentyl phthalate	DPP	C6H4[COO(CH2)4CH3]2
5	Diisopentyl phthalate	DiPP	C6H4[COO(CH2)3CH(CH3)2]2
6	Bis(butoxyethyl) phthalate	BBOP	C6H4[COO(CH2)2O(CH2)3CH3]2
6	Dicyclohexyl phthalate	DCHP	C6H4[COOC6H11]2
6	Di-n-hexyl phthalate	DNHP	C6H4[COO(CH2)5CH3]2
7	Diheptyl phthalate	DHP	C6H4[COO(CH2)4CH(CH3)2]2
8	Di(2-ethylhexyl) phthalate	DEHP	C6H4[COOCH2CH(C2H5)(CH2)3CH3]2
8	Di(n-octyl) phthalate	DNOP	C6H4[COO(CH2)7CH3]2
9	Diisononyl phthalate	DINP	C6H4[COO(CH2)6CH(CH3)2]2
10	Diisodecyl phthalate	DIDP	C6H4[COO(CH2)7CH(CH3)2]2
11	Diundecyl phthalate	DUP	C6H4[COO(CH2)10CH3]2
13	Ditridecyl phthalate	DTDP	C6H4[COO(CH2)12CH3]2

### Phthalate Metabolism

Following ingestion, lipases in the intestinal epithelium, liver, and other tissues hydrolyze diesters of phthalates to its monoester derivatives (Albro et al., 1982). For example, DEHP is metabolized into monoester (MEHP), which is then widely distributed in the body. These monoesters can be further metabolized into soluble metabolites. Such as DEHP, after its conversion to MEHP, it is metabolized into mono (2-ethyl-5-hydroxyhexyl) phthalate and mono (2-ethyl-5-oxohexyl) phthalate (Sugawara and Sugawara, 1986), which together with MEHP is secreted into the urine. Study also showed that almost 98% urinary samples of pregnant Brazilian women had diisopentyl phthalate (DIPP) metabolite, monoisopentyl phthalate and two additional secondary oxidized metabolites, 3OH-monoisopentyl phthalate and 4OH-monoisopentyl phthalate (Suzuki and Yoshida, 1978). For DBP, MBP is always measurable in the meconium of newborns (Zhang et al., 2009b).

### Phthalate Effects and Human Epidemiological Studies

Increased human evidence shows that phthalates have adverse effects on Leydig cell development, thus causing male reproductive tract anomalies (cryptorchidism, hypospadias, and shortened anogenital distance) and disrupted spermatogenesis (low sperm counts and sperm motility). The disrupted spermatogenesis could be caused by the prenatal exposure to the phthalates (Fisher et al., 2003; Sharpe, 2003; Ge et al., 2007; Mahood et al., 2007; Hu et al., 2009), as the disease referred as the testicular dysgenesis syndrome (Fisher et al., 2003).

A study with 270 cryptorchidism patients, 75 hypospadias patients, and 300 control subjects showed that DEHP metabolite levels in the amniotic fluid were negatively associated with human male fetal gonadal function (Jensen et al., 2015). Considering the DINP metabolite, Jensen et al. cannot exclude an association of DEHP with hypospadias and less strongly with cryptorchidism (Jensen et al., 2015). A cohort with 243 control subjects, 227 cryptorchids (both bilateral and unilateral ones), and 73 hypospadias patients in Danish showed that amniotic fluid INSL3 levels during the critical time window in human pregnancy were related to cryptorchidism and hypospadias and were negatively affected by phthalates (Suzuki, 1980). A small cohort with 50 couples in an IVF center to investigate the effects of parental exposure to phthalates on early embryo development found that there was a negative association between the concentrations of DEHP metabolite (MEHP) in males and embryo development (Suvanto and Kormano, 1970). However, a Danish-Finnish cohort study on cryptorchidism with 62 cryptorchid and 68 healthy boys from 1997 to 2001 showed that no association between phthalate monoester levels and cryptorchidism but positive correlations of MEP and MBP with SHBG, correlations of MMP, MEP, and MBP with LH: free testosterone ratio, and MINP with LH as well as negative correlation of MBP with free testosterone (Main et al., 2006).

A meta-analysis of 5 observational prospective cohort studies demonstrated that the increase in maternal urinary levels of DEHP metabolites was associated with the reduction of baby male anogenital distance, a biomarker for reduction of androgen production by Leydig cells (Suzuki et al., 1998). However, in a cohort (245 mothers of boys) with urinary concentrations of 12 phthalate metabolites of diethyl, di-n-butyl, diisobutyl, di(2-ethylhexyl), butylbenzyl, and diisononyl phthalate at about week 28 showed no significant trends towards shorter anogenital distance in boys with higher phthalate exposures in this low exposed Danish population (Jensen et al., 2016). Sathyanarayana et al. studied the urinary phthalates and birth outcome in 371 subjects from 2010 to 2012 and also demonstrated the negative association of phthalates and genital anomaly (Sathyanarayana et al., 2016).

A cohort with 796 male students also demonstrated that phthalate exposure was associated with bad semen quality and lower androgen levels in men even with a dose given below the U.S. EPA reference doses (Sugiura et al., 2005). A study about 253 male partners of subfertile and 37 fertile couples in a southern Taiwan hospital showed that the Leydig cell biomarker INSL3 and androgen were negatively associated with phthalates, including DEHP and its major metabolite MEHP (Sunderman, 1987). A study with 379 men in the infertility clinic in the U.S. showed that sperm DNA damage was associated with mono-ethyl phthalate (MEP) and DEHP metabolites (Hauser et al., 2007). However, a study with 349 men analyzed the relationship between DEHP metabolites and semen concentration and motility and did not find any association (Sutherland et al., 1974). Interestingly, a large case-control study for amniotic fluid INSL3 during the critical time window in human gestation showed that cryptorchidism and hypospadias are significantly related to increase in amniotic INSL3 during gestational weeks 13–16 and irrespective of cryptorchidism or not INSL3 was indeed negatively correlated with phthalate loads (Anand-Ivell et al., 2018).

A cohort with 913 men aged 20–22 years in the Western Australian were performed for prenatal serum phthalate metabolite in maternal sera collected at 18 and 34 weeks gestation and testicular volume, semen analysis, and serum concentrations of gonadotrophins, inhibin B, and testosterone and it showed that metabolites of DEHP and DINP were negatively correlated testicular volume (Hart et al., 2018). There was little evidence of associations between urinary phthalate metabolites or sums of phthalates with reproductive hormones or semen quality (Joensen et al., 2012). A Chinese cohort with semen (n = 687) and blood samples (n = 342) in the male partners of sub-fertile couples in Wuhan China showed that semen phthalate metabolites (MBP and DEHP metabolites) were significantly associated with decreases in semen volume and that sperm curvilinear velocity with MBzP and MEHP (Wang et al., 2016).

### Effects of Phthalates on Fetal Leydig Cell Development and Reproductive Tract Development

During development, testosterone binds to androgen receptors and stimulates differentiation of the vas deferens, epididymis, and seminal vesicles from the embryonic Wolffian duct. Development of the prostate and virilization of the external genitalia, tissues that are derived from the caudal portions of the embryonic urogenital sinus, are also androgen-dependent (Capel, 1998). It is hypothesized that the earlier in development a lesion is inflicted, the greater the likelihood of its persistence into adulthood (Cooper and Kavlock, 1997). Gestational exposure to a number of phthalates, including DEHP, DBP, BBP, DiPP, DHP, and DINP suppressed sexual differentiation as evidenced by reduced anogenital distance and retention of female-like areolas/nipples as well as reduction of testosterone levels in the testis in male pup rats (Suzuki and Yoshida, 1978; Syed et al., 1997; Foster et al., 2001; Saillenfait et al., 2013; Kilcoyne et al., 2014; Gray et al., 2015; Lara et al., 2017). Anogenital distance and penile-prepuce separation represent non-invasive indicators of androgen status in prepubertal rats (Foster et al., 2001). Moreover, affected adults exhibit epididymal agenesis and reduced sex accessory gland weights (Gray et al., 2000). These observations are similar to those reported in studies of chemicals that interfere with androgen receptor function. For example, the major and persistent DDT (1,1,1-trichloro-2, 2-bis (p-chlorophenyl) ethane) metabolite p, p′-DDE (1,1-dichloro-2, 2-bis (p-chlorophenyl) ethylene), considered a prototype antiandrogen, inhibits androgen binding to androgen receptors and androgen-induced transcriptional activity, thereby suppressing androgen action in developing pubertal and adult male rats (Kelce et al., 1991). Similarly, exposure of rodents to androgen receptor antagonists vinclozolin and procymidone inhibited fetal testicular androgen biosynthesis and demasculinized neonates (Gray et al., 1999). Thus, DEHP and other compounds that interfere with androgen receptor-mediated activity are of epidemiological significance given their ubiquitous presence in the environment. Many data show that fetal Leydig cells are the primary target for phthalate action. Evidence that androgen action is an essential requirement for reproductive tract development and function is seen in human males with a mutation in the androgen receptor gene. These individuals exhibit complete androgen insensitivity syndrome and develop external and internal female genitalia (Patterson et al., 1994). Genetic males (XY karyotype), in fact, develop a female phenotype in the absence of a functional androgen receptor protein (Wilson, 1989). Interestingly, many phthalates, including DEP (Hu et al., 2018), DBP (Mahood et al., 2005; Hallmark et al., 2007; Van Den Driesche et al., 2012), DCHP (Li et al., 2016), DEHP (Lin et al., 2008b; Hu et al., 2018), and DINP (Li et al., 2014) cause the fetal Leydig cell aggregation. Although the exact mechanism of the causing factors after prenatal phthalate exposure remains unclear, the increased secretion of some growth factors and cytokines such as leukemia inhibitory factor might be involved (Lin et al., 2008a). The fetal Leydig cell aggregation could lead to the delay of the postnatal Leydig cell population in rats after in utero treatment of DBP (Chen et al., 2017).

### Effects of Phthalates on Fetal Leydig Cell Steroidogenesis and INSL3 Secretion

Many studies are performed for various phthalates in the regulation of *Insl3* and steroidogenesis-related gene and protein expression. Indeed, many phthalates down-regulated *Insl3* and several steroidogenesis-related gene and proteins. Lin et al. reported that DEHP dose-dependently down-regulated expression of *Lhcgr*, *Scarb1*, *Star*, *Cyp11a1*, and *Insl3* and some their proteins (Lin et al., 2008a). Hannas et al. also reported that a dose-dependent down-regulation of *Star*, *Cyp11a1*, *Insl3* and reduction of testosterone production in fetal Leydig cells after in utero DEHP expression (Thomas et al., 1987; Borch et al., 2006b; Stroheker et al., 2006; Hannas et al., 2011). DPrP, DCHP, DBP, DiBP, DPP, and DINP had the similar actions (Takkar et al., 1968; Taylor, 1976; Thompson, 1976; Tam and Liu, 1985; Lehmann et al., 2004; Mckinnell et al., 2005; Borch et al., 2006a; Takiguchi and Yoshihara, 2006; Lin et al., 2008b; Thompson and Bannigan, 2008; Li et al., 2014; Li et al., 2016; Hu et al., 2018). Individual phthalates with a similar mechanism of action exert cumulative and dose additive effects on fetal testosterone production and fetal Leydig cell aggregation when they were exposed in a mixture (Howdeshell et al., 2008; Saillenfait et al., 2011; Hu et al., 2018). Interestingly, DMP and DEP as well as phthalates with 10 carbons (such as dioctyl tere-phthalate) in the alcohol moiety had almost no effects on testosterone production (Gray et al., 2000; Liu et al., 2005). Many phthalate diesters could be metabolized into mono-phthalates to exert action. Indeed, reports showed that MBP (the metabolite of DBP) and MEHP (the metabolite of DHEP) showed similar potency to down-regulate *Insl3* expression in rats (Imajima et al., 1997; Li and Kim, 2003; Terada et al., 2005).

Although the exact mechanism(s) of phthalates on fetal Leydig cell function are still not well understood, several studies suggest that phthalates can regulate fetal Leydig cell function *via* several signaling pathways. Phthalates do not bind to androgen receptors *via* receptor binding assay, and they exert actions possibly *via* inhibiting androgen biosynthesis (Hu et al., 2009). Other possible mechanisms are that phthalate metabolites bind to peroxisome proliferator-activated receptors (PPAR) (Corton and Lapinskas, 2005). Gene expression studies suggest that phthalates (e.g. DBP) affect steroidogenic gene expression *via* indirect suppression of NR5A1 action in fetal rat testes (Kuhl et al., 2007; Plummer et al., 2007). DBP inhibits NR5A1 binding in the promoters and introns of NR5A1-transactivated genes (such as *Cyp11a1*, *Cyp17a1*, and *Star*) that are down-regulated by DBP (Plummer et al., 2013). The binding of NR5A1 to the NR5A1-transactivated gene promoter of *Fshr* is not affected phthalates (Kuhl et al., 2007; Plummer et al., 2007; Plummer et al., 2013), conforms to the unaltered *Fshr* expression after the treatment of phthalates (DEHP, DBP, and DINP) (Kuhl et al., 2007; Plummer et al., 2007; Lin et al., 2008a; Plummer et al., 2013; Li et al., 2014), therefore suggesting that the effects of phthalates on NR5A1 binding are indirect. The suppressive actions of phthalates on the binding of NR5A1 to the NR5A1-mediated steroidogenic genes correlate with profiles in the binding of PPARα, a subtype nuclear receptor of PPAR (Plummer et al., 2013). Phthalates have been shown to activate PPARα (Hurst and Waxman, 2003). It has been demonstrated that PPARα is involved in the suppression of Leydig cell steroidogenesis by Leydig cells both *in vitro* (Gazouli et al., 2002) and *in vivo* (Corton and Lapinskas, 2005). 2005). Indeed, the inhibition of NR5A1-transactivated steroidogenic genes by DBP is correlated to the increases in PPARα binding (Plummer et al., 2013). However, this increases in PPARα binding to steroidogenic genes happen at binding sites different from those of NR5A1 (Plummer et al., 2013), suggesting that PPARα might be an indirect repressor of NR5A1 binding. PPARα and NR5A1 share a common coactivator, CREB-binding protein (CBP), which has limited levels (Mccampbell et al., 2000), and binding of CBP to PPARα could compete with NR5A1 for its transactivation functions. CBP can form a transcriptional complex with PPARα for PPARα transactivation (Misra et al., 2002).

### Effects of Phthalates on Stem Leydig Cell Function

There is growing evidence to show that several phthalates affect stem Leydig cell function, thus affecting Leydig cell development during fetal and postnatal period. Indeed, DEHP increased Leydig cell number after prepubertal treatment (Akingbemi et al., 2004) possibly *via* increasing LH and estradiol secretion, which acting on stem Leydig cells and their progenitors to up-regulate *Ccnd3* expression (Akingbemi et al., 2004). DEHP also increased Leydig cell number in an ethane dimethane sulfonate-treated Leydig cell regeneration model, possibly promoting the proliferation of stem Leydig cells and rapid commitment into progenitors (Li et al., 2012; Guo et al., 2013). DBP also has the similar action to increase Leydig cell number in ethane dimethane sulfonate-treated model (Heng et al., 2011).

## Conclusion

Roles of phthalates in the regulation of the development of fetal Leydig cells were discussed. Mostly, phthalates adversely affect fetal Leydig cell development and function. The high levels of phthalate exposure could impair fetal Leydig cell function, thus causing lower androgen and INSL3 production and subsequent of the reproductive tract anomalies in new-born baby boys (**Figure 1**). There is a clear structure activity response between different phthalates for the inhibition of testosterone by fetal Leydig cells, with phthalates of 3–9 carbon atoms in the carbon chain being active and those of 1–2 or 10 or higher carbon atoms in the carbon being inactive.

## Author Contributions

Conceived and designed the review: ZL, LL, and R-SG. Literature research: YW, CN, XL, and QZ. Table and figures design: YW and CN. Wrote the review: YW, CN, and R-SG. Review final version approval: ZL, LL, and R-SG.

## Conflict of Interest

The authors declare that the research was conducted in the absence of any commercial or financial relationships that could be construed as a potential conflict of interest.
